# On the Estimation Accuracy of the 3D Body Center of Mass Trajectory during Human Locomotion: Inverse vs. Forward Dynamics

**DOI:** 10.3389/fphys.2017.00129

**Published:** 2017-03-08

**Authors:** Gaspare Pavei, Elena Seminati, Dario Cazzola, Alberto E. Minetti

**Affiliations:** ^1^Laboratory of Physiomechanics of Locomotion, Department of Pathophysiology and Transplantation, University of MilanMilan, Italy; ^2^Sport, Health and Exercise Science, Department for Health, University of BathBath, UK

**Keywords:** ground reaction forces, kinematics, mechanical work, walking, running, race walking, skipping

## Abstract

The dynamics of body center of mass (BCoM) 3D trajectory during locomotion is crucial to the mechanical understanding of the different gaits. Forward Dynamics (FD) obtains BCoM motion from ground reaction forces while Inverse Dynamics (ID) estimates BCoM position and speed from motion capture of body segments. These two techniques are widely used by the literature on the estimation of BCoM. Despite the specific pros and cons of both methods, FD is less biased and considered as the golden standard, while ID estimates strongly depend on the segmental model adopted to schematically represent the moving body. In these experiments a single subject walked, ran, (uni- and bi-laterally) skipped, and race-walked at a wide range of speeds on a treadmill with force sensors underneath. In all conditions a simultaneous motion capture (8 cameras, 36 markers) took place. 3D BCoM trajectories computed according to five marker set models of ID have been compared to the one obtained by FD on the same (about 2,700) strides. Such a comparison aims to check the validity of the investigated models to capture the “true” dynamics of gaits in terms of distance between paths, mechanical external work and energy recovery. Results allow to conclude that: (1) among gaits, race walking is the most critical in being described by ID, (2) among the investigated segmental models, those capturing the motion of four limbs and trunk more closely reproduce the subtle temporal and spatial changes of BCoM trajectory within the strides of most gaits, (3) FD-ID discrepancy in external work is speed dependent within a gait in the most unsuccessful models, and (4) the internal work is not affected by the difference in BCoM estimates.

## Introduction

However, complex a form of terrestrial gait may seem, locomotion is ultimately the translation of the body center of mass (BCoM) through space. This occurs because part of the generated muscle force causes ground reaction forces that, directed toward BCoM, make us move. The dynamic 3D trajectory of BCoM, when reliably measured, directly reflects the role of important biomechanical and physiological variables as muscle activity, mechanical work done (Fenn, 1930; Cavagna et al., 1963), the energy saving strategies (Heglund et al., 1982; Minetti, 1998a), and, indirectly, contributes to explain the determinants of metabolic consumption (Minetti et al., 1993). Nowadays, a detailed analysis of 3D BCOM trajectory is potentially useful to detect physiological and pathological right/left asymmetries of locomotion. An accurate reconstruction of the 3D path of BCoM (3D Lissajous contour in local coordinates, Minetti et al., 2011a) could help the diagnosis and treatment, including prostheses adaptation, of gait impairments.

Two methodological approaches are available, depending on the investigated signal and its role in the mechanical cascade of events: (1) net ground reaction forces are sampled through dynamometric platforms, integrated over time to obtain BCoM speed and again to asses BCoM displacement, and (2) the 3D kinematics of body joints are captured, the centers of mass of body segments are computed, and BCoM trajectory is finally obtained as a weighed mean of body segments' positions. Since the (literally) *primum movens* in mechanics is force, it is straightforward to name the first technique as “forward dynamics” (FD, from force to body energy related to its speed and position) and the second one as “inverse dynamics” (ID, from spatial coordinates to displacement, speed, and energy).

Although FD could be, even intuitively, considered the reference standard (see below), neither of the two methodologies prevailed since the very early experiments in locomotion biomechanics (Braune and Fischer, 1895; Fenn, 1930; Elftman, 1939), due to the pros and cons of each technique.

FD has the great advantages that no assumptions are needed: (i) about the anthropometry of the investigated subject/animal, and (ii) about the rigidity of body segments (actually force signals also capture whichever wobbling mass oscillation, as for abdominal viscera; Minetti and Belli, 1994); in addition, (iii) data processing involves integrations, which inherently smooth the noise of the signal, requiring just a “shallow” filtering. But: (a) the instrumental cost limits the number of available force platforms and precludes a sampling longer than a few steps, particularly at high speed, (b) the results depends on the initial conditions (three initial speeds and three initial spatial locations, i.e., six integration constants), which should be separately measured (e.g., progression speed obtained from photocells), (c) being confined to a short walkway, steady state requirements in terms of the same average speed of the performed strides is seldom met, (d) a drift in force signals (0 and body weight values) can occur during long lasting acquisitions with (piezoelectric based, especially) dynamometric platforms, or with IMU based motion capture systems (due to alignment issues of the sensors), where the double integration of the drifted signal can lead to biased results.

Conversely, ID: (i) allows the use of treadmills, where speed is controlled and kept constant, (ii) provides the opportunity to sample hundreds of strides, whose average values will more reliably reflect the “typical” gait pattern, (iii) initial conditions are more easily approximated (e.g., constant speed, BCoM absolute height). But: (a) the assumptions about anthropometry (body size, conformation, gender, age, etc.) and (b) the rigidity of body segments, crucial to the accuracy of BCoM position, is only approximated due to skin motion of adhesive markers (Leardini et al., 2005), (c) different schematic models of body segments, chosen to capture the essence of movement and to estimate BCoM position, could lead to very different estimates, (d) markers on body joints location only approximate the “true” pivotal point (Chiari et al., 2005), (e) the motion of wobbling masses, e.g., muscles and viscera within the trunk (Minetti and Belli, 1994; Zelik and Kuo, 2010; Cazzola et al., 2014), which can be remarkable in obese subjects, cannot be accurately measured and (f) data processing involves differentiation, which inherently enhances the noise of the signal, which needs a substantial filtering to avoid unrealistic peaks, say, in BCoM kinetic energy.

For the above reasons, FD and ID are expected to provide different results when analysing the same subject during locomotion. The amount of this difference has been investigated in the literature, mainly regarding the BCoM vertical excursion, at one walking speed (Thirunarayan et al., 1996; Whittle, 1997; Eames et al., 1999; Gard et al., 2004; Gutierrez-Farewik et al., 2006) and in a wide range of running speeds (Gullstrand et al., 2009). The comparison has been pursued by simultaneously using dynamometric platform and 3D motion analysis on a short walkway. It has been suggested to combine FD and ID to further improve some disadvantages of both methods (Maus et al., 2011). However, at present, most of the laboratories use either FD or ID methods. Also in the combined method the best marker set for the ID part still need to be established.

The biomechanics of locomotion nowadays extends well-beyond walking and running. Skipping (Minetti, 1998a), race walking (Pavei et al., 2014), backward, lateral, and circular locomotion (Cavagna et al., 2011; Minetti et al., 2011b; Handford and Srinivasan, 2014) are also part of the usual gait repertoire, particularly in sport activities.

This paper originates from a preliminary experiment on race walking, where a remarkable discrepancy between BCoM trajectory obtained from motion analysis (ID) and dynamometric platforms (FD) was observed (Pavei et al., 2012). We hypothesized that the rigid segment modeling adopted did not capture the essence of the complex body movement in race walking, where the trunk and pelvis twist and basculate with a complex pattern. This was the motivation to start a much more comprehensive study, where many gaits, and speeds could be simultaneously analyzed in terms of BCoM trajectory and related parameters estimated by FD and ID on the same stride, with the aim of checking for precision and accuracy of the ID estimation using different models.

For this purpose data should be collected from the same subject in the same session, possibly in a substantial number of strides. To fulfill all these requirements, a more modern experimental setup can be used: 3D motion capture + instrumented treadmills with force sensors, from prototypal level (Kram et al., 1998) to commercially available versions (e.g., Arsalis) mainly produced to estimate net joint moments.

## Materials and methods

### Experimental protocol

One subject (1.78 m height, 63 kg body mass, 26 years old), skilled with treadmill locomotion and trained to all gaits was asked to perform walking, running, skipping and race walking trials at a wide range of speeds. The study was approved by the University Ethics Committee and the subject signed an informed consent before the experimental test in accordance with the Declaration of Helsinki.

The protocol included: walking at speed range of 0.56–1.94 m s^−1^ (0.28 m s^−1^ increment); running at 2.22–5.56 m s^−1^ (0.28 m s^−1^ increment); race walking at 2.22–4.17 m s^−1^ (0.28 m s^−1^ increment); skipping (uni- and bi-lateral) at 0.83–3.06 m s^−1^ (0.56 m s^−1^ increment). At each speed data were collected for 1 min after the subject reached a steady locomotion and 3 min of rest elapsed between consecutive acquisitions. Due to the specific aims of the study, i.e., the comparison between methods (ID vs. FD) in estimating biomechanical variables (as the BCoM trajectory or external work) on the same strides, we extended the number of measurements (37 sessions, for a total of about 2,700 strides) and restricted the participant sample size to one, as to privilege an intra-subject analysis approach.

### Data acquisition

Kinematic data were collected by means of a 8-camera Vicon system (Oxford Metrics, UK) at a sampling rate of 300 Hz. A Mercury LT med treadmill (HP Cosmos, Germany) with a 1.5 m long and 0.5 m wide belt, equipped with four 3D strain-gauge force traducers (Dierick et al., 2004) was used to collect ground reaction forces at 900 Hz. Analyses were performed stride by stride, and a threshold on the vertical position of the left heel marker, with respect to the belt, was used to detect heel strike. Experiments were carried out at the “Unit Laboratory of Physiology and Biomechanics of Locomotion” (LOCO) at the Universitè Catholique de Louvain (Belgium).

### Data analysis

Five different kinematic models (ID) for BCoM calculation were used: (i) a single marker (e.g., Belli et al., 1993) placed on the spinal process of 7^th^ vertebra (C7), (ii) the “mean iliac spines” obtained by the position of four markers placed on the anterior and posterior iliac spines (Spinae) (Whittle, 1997), (iii) a 11-segment body: trunk, arms, forearms with hands, thighs, shanks, and feet modeled with 18 markers, based on Dempster tables (18 mkr; Minetti et al., 1993), (iv) a 14-segment body: head, upper middle and lower trunk, arms, forearms with hands, thighs, shanks, and feet modeled with 22 markers, based on De Leva tables (De Leva) (de Leva, 1996), (v) a 14-segment body: head, trunk, arms, forearms, hands, thighs, shanks and feet modeled with 36 markers, as described in Vicon Plug-in-Gait model (PlugInGait) (Nexus 1.81 version, Oxford Metrics UK). For segments endpoints see Table 1.

**Table 1:** **Segments' endpoints of each kinematic model**.

	**18 mkr**	**De leva**	**PlugInGait**
Arm	Glenohumeral axis–elbow axis	Glenohumeral axis–elbow axis	Acromion–elbow axis
Forearm	Elbow axis–ulnar styloid^a^	Elbow axis–ulnar styloid	Elbow axis–radius and ulnae epicondyles
Hand	//	Ulnar styloid–dorsum of hand	Radius and ulnae epicondyle–dorsum of hand
Thigh	Great trochanter–femoral condyle	Great trochanter–femoral condyle	Anterior and posterior iliac spine–femoral condyle
Shank	Femoral condyle–malleolus	Femoral condyle–malleolus	Femoral condyle–malleolus
Foot	Heel–5^th^ metatarsal	Calcaneous–toe	Calcaneous–toe
Trunk	Glenohumeral axis–great trochanter^b^ (right and left sides separated)	Upper Trunk: Spinous process of C7—middle point of two markers placed laterally (right and left) omphalion Middle Trunk: middle point of two markers placed laterally (right and left) to omphalion—middle point of two markers placed laterally (right and left) to xyphion Lower Trunk: middle point of two markers placed laterally (right and left) to xyphion—middle point of right and left great trochanter	Spinous process of C7 and T10 vertebrae, sternum, jugular notch, and bilaterally acromion and anterior and posterior iliac spines
Head	//	Vertex–spinous process of C7	Temple and a marker symmetrical on the back of the head

*Markers are bilateral when describing two sides' limbs*.

a *Include also hand*.

b *Include also head*.

Markers position of C7, Spinae, 18 mkr and De Leva were filtered through a “zero-lag” second order Butterworth low pass filter with a cut-off frequency detected by a residual analysis on each marker coordinate (Winter, 1979). Conversely, PlugInGait was already filtered through a Nexus software routine (Vicon, Oxford Metrics, UK).

**Figure 1:**
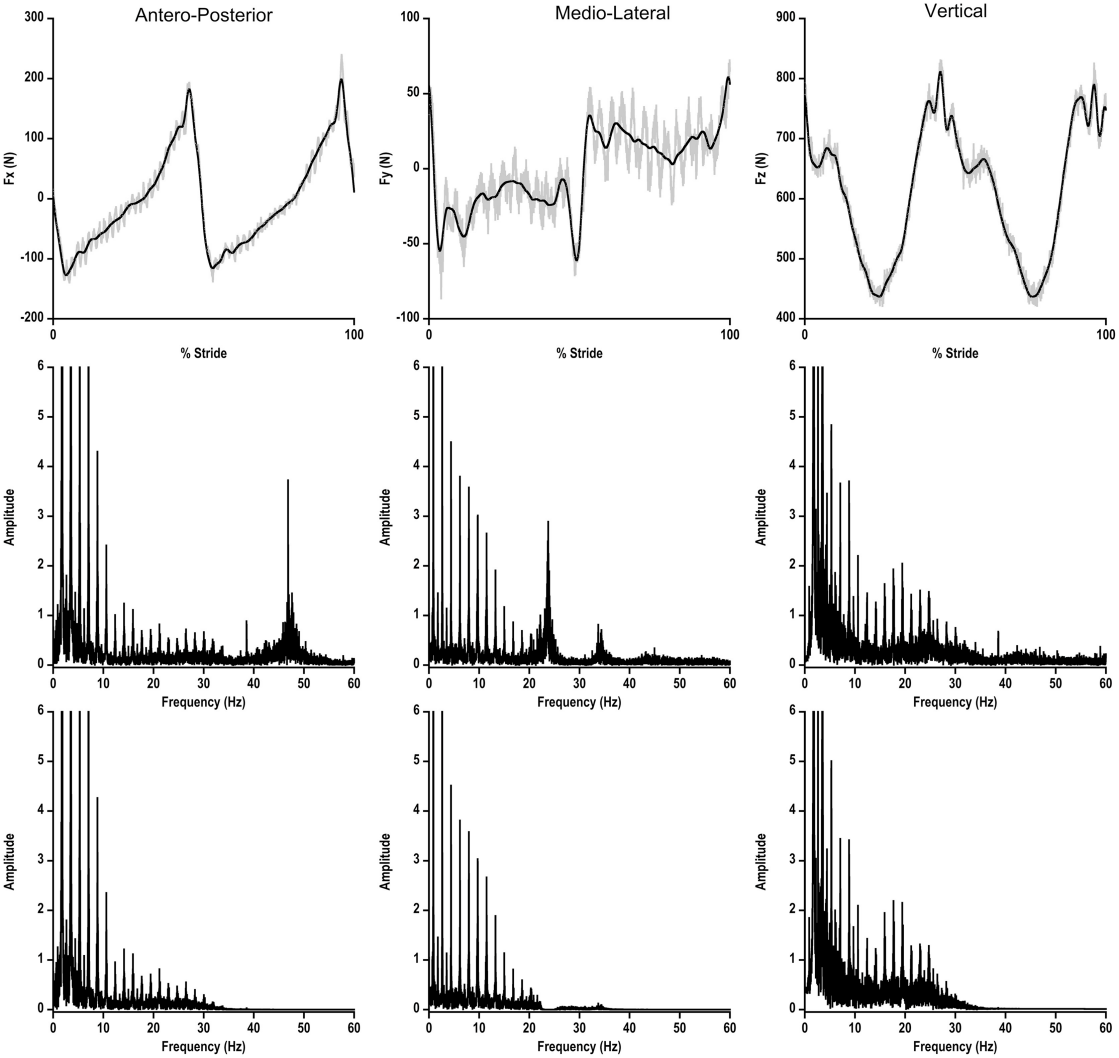
**Upper Panel:** ground reaction force in the three planes of motion during one walking stride at 1.39 m s^−1^ on the instrumented treadmill. Gray line represents the original signal, black line represents the filtered signal. **Lower Panel**: power spectrum of each ground reaction force before (upper part) and after (lower part) the filtering process.

Differently, ground reaction forces (FD) were filtered both for noise reduction purposes and to allow a subsequent BCoM displacement description based on a Fourier Series with six harmonics (Minetti et al., 2011a). The spectral analysis, showed peaks of noise frequencies at 39, 47, 110, and 114 Hz, which were speed and gait independent, likely induced by the treadmill framework and engine vibrations. Force traces were filtered through a forward and reverse low pass, 4^th^ order Butterworth filter with a cut off frequency of 30 Hz (just above Nyquist frequency). In order to delete an additional component at 24 Hz in the medio-lateral force, a 3^rd^ order Bessel notch filter set at 24 Hz with a “stopband attenuation” of 60 dB was used (Figure 1).

The filtered signal of ground reaction forces was split in each single stride, and all the subsequent processing activities were performed over each stride.

BCoM position from ground reaction forces was computed by double integration according to Cavagna (1975) and integration constants were calculated as described in appendix (Saibene and Minetti, 2003) (in brief, initial speed constants are reset according to the deviation from expected average values, as caused by starting the integration in a time frame where speed assumptions do not hold). The obtained BCoM trajectory was then down sampled (1/3) in order to match inverse dynamics data length (in figures and tables this method is labeled as GRF) for each stride. The obtained single stride BCoM trajectories, with the same sample size from FD and ID models, were forced to become closed loops (Minetti et al., 2011a). To be able to mathematically describe BCoM 3D trajectories, the combined techniques are “Fourier Analysis” and “Lissajous 3D Contours.” Both techniques assume that whichever trajectory in space will return, after one complete cycle, exactly to the start position. This never happens in locomotion, although the distance between start and end positions is normally quite small. In the developed method, the end each stride loop is forced to correspond to the start by filling the gap (in 3D) progressively along the stride cycle (Minetti et al., 2011a). Average 3D coordinates were subtracted from ID contours to make them centered on (0, 0, 0; Minetti et al., 2011a) and comparable to FD by using a point-by-point 3D root mean square (3D RMS, m). In order to avoid any possible phase shift introduced by filtering, an automatic 3D routine time-shifted ID loops in order to find the minimal 3D RMS. Based on preliminary experiments showing different 3D excursions of BCoM for the different gaits, we decided also to provide a dimensionless 3D RMS value, i.e., standardized for space occupancy in terms of the cubic root of the volume occupied by the contour obtained via FD (= 3D RMS · volume of contour ^−1/3^), in order to express the trajectory match between FD and ID independently from their size.

The (positive) external mechanical work (W_EXT_, J kg^−1^ m^−1^), which is defined as the work done to accelerate and raise BCoM, was calculated for each methodology by summing the increments of total energy (= potential + kinetic energy) time course (Cavagna and Margaria, 1966). Energy Recovery, the ability to exchange potential and kinetic energy as in an ideal pendulum, was also calculated (Cavagna et al., 1976). Mechanical internal work (W_INT_, J kg^−1^ m^−1^), i.e., the work done to accelerate body segments with respect to BCoM (Cavagna and Kaneko, 1977; Minetti, 1998b), was calculated in two different ways, in order to investigate potential differences in the two methods: the movement of body segments (18 mkr model) with respect to: (a) ID obtained BCoM (18 mkr model) and (b) to GRF BCoM (FD).

### Statistics

A one-way ANOVA for repeated measures was used to pairwise compare, for each stride obtained by FD and by each of ID methods, W_EXT_ and Energy Recovery, at different speeds and gaits. The significant level was set at α = 0.05. In this comparison, sample size is the number of strides, which (pairwise) varies according to stride frequency (gait type and speed), ranging from 40 to 82. Statistical analyses were performed with SPSS v20 (IBM).

## Results

**Figure 2:**
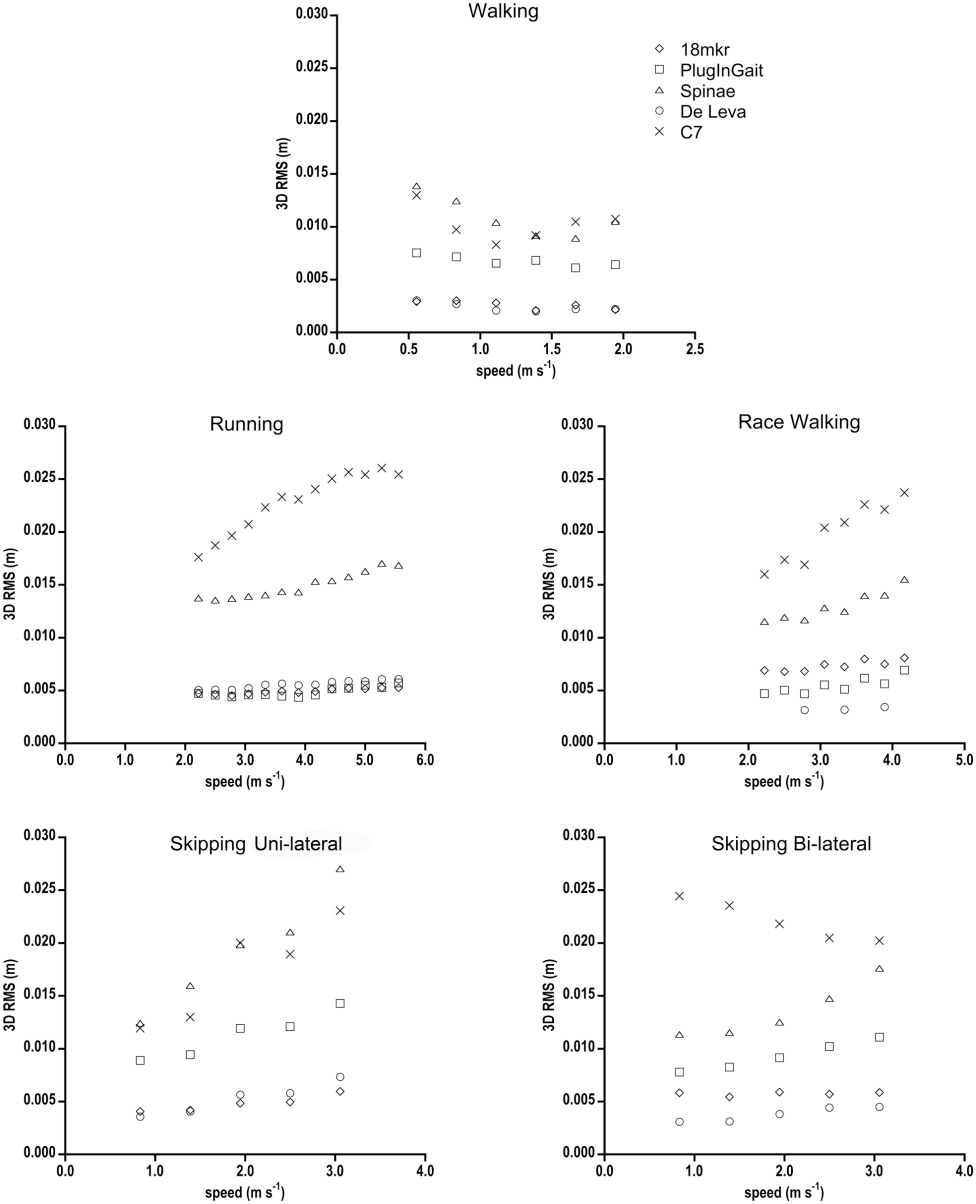
**3D RMS (m) between BCoM trajectories from GRF and from each Inverse Dynamic model, as a function of speed for all gaits**. Standard deviations have been omitted for clarity. Due to technical problems, race walking RMS data for the De Leva model refer just to three speeds.

The 3D RMS (m) between GRF and each Inverse Dynamics model for every gait at increasing speeds is presented in Figure 2. C7 and Spinae showed the greatest bias with respect to GRF, whereas 18 mkr and De Leva well-matched all gaits, with the exception of race walking for 18 mkr. PlugInGait was in accordance to Forward Dynamics when used for running and race walking, quite far in the other gaits (see Table 2 for percentage differences in values). In the successive figures the reference data (FD method) are labeled as GRF and shown as filled circles, while the ID methods are represented by lighter symbols for sake of an easy comparison.

**Table 2:** **Mean differences (±***SD***) between ID and FD values of the relevant variables, for each investigated gaits; all speeds are averaged for each condition**.

		**Model**				
		**PlugInGait**	**De Leva**	**18 mkr**	**Spinae**	**C7**
*n*° markers		36	22	18	4	1
3D RMS (mm)	Walking	6.66 ± 0.50	2.54 ± 0.54	2.63 ± 0.61	10.71 ± 1.66	10.32 ± 1.46
	Running	4.86 ± 0.45	5.02 ± 0.47	6.78 ± 0.41	14.93 ± 1.23	22.86 ± 2.84
	Skipping Unilateral	11.32 ± 2.18	3.79 ± 0.68	4.83 ± 1.01	19.25 ± 5.51	17.39 ± 4.75
	Skipping Bilateral	9.29 ± 1.36	5.28 ± 1.50	4.64 ± 0.39	13.54 ± 2.64	22.10 ± 1.85
	Race Walking	5.54 ± 0.73	3.26 ± 0.17	8.11 ± 0.84	13.08 ± 1.34	20.33 ± 2.65
W_EXT_	Walking	+13 ± 35%	+13 ± 11%	+14 ± 10%	+37 ± 16%	+16 ± 19%
	Running	+14 ± 14%	+10 ± 2%	+23 ± 7%	+110 ± 39%	−19 ± 6%
	Skipping Unilateral	−34 ± 11%	+12 ± 5%	+15 ± 4%	+35 ± 22%	+4 ± 8%
	Skipping Bilateral	+11 ± 13%	+5 ± 1%	+10 ± 5%	+20 ± 10%	+8 ± 7%
	Race Walking	−16 ± 22%	+18 ± 3%	+53 ± 7%	+79 ± 8%	−26 ± 14%
Energy recovery	Walking	−21 ± 22%	−7 ± 6%	−8 ± 5%	+2 ± 6%	−21 ± 12%
	Running	+759 ± 169%	+285 ± 74%	+769 ± 146%	+626 ± 170%	+970 ± 140%
	Skipping Unilateral	−34 ± 11%	−17 ± 4%	−14 ± 3%	+22 ± 10%	+20 ± 9%
	Skipping Bilateral	−24 ± 7%	−2 ± 1%	−7 ± 8%	+22 ± 14%	−32 ± 5%
	Race walking	+1332 ± 714%	+46 ± 102%	+423 ± 341%	+20 ± 126%	+1152 ± 587%

*W_EXT_ and Energy Recovery mean values are expressed as (ID-FD)/FD %*.

**Figure 3:**
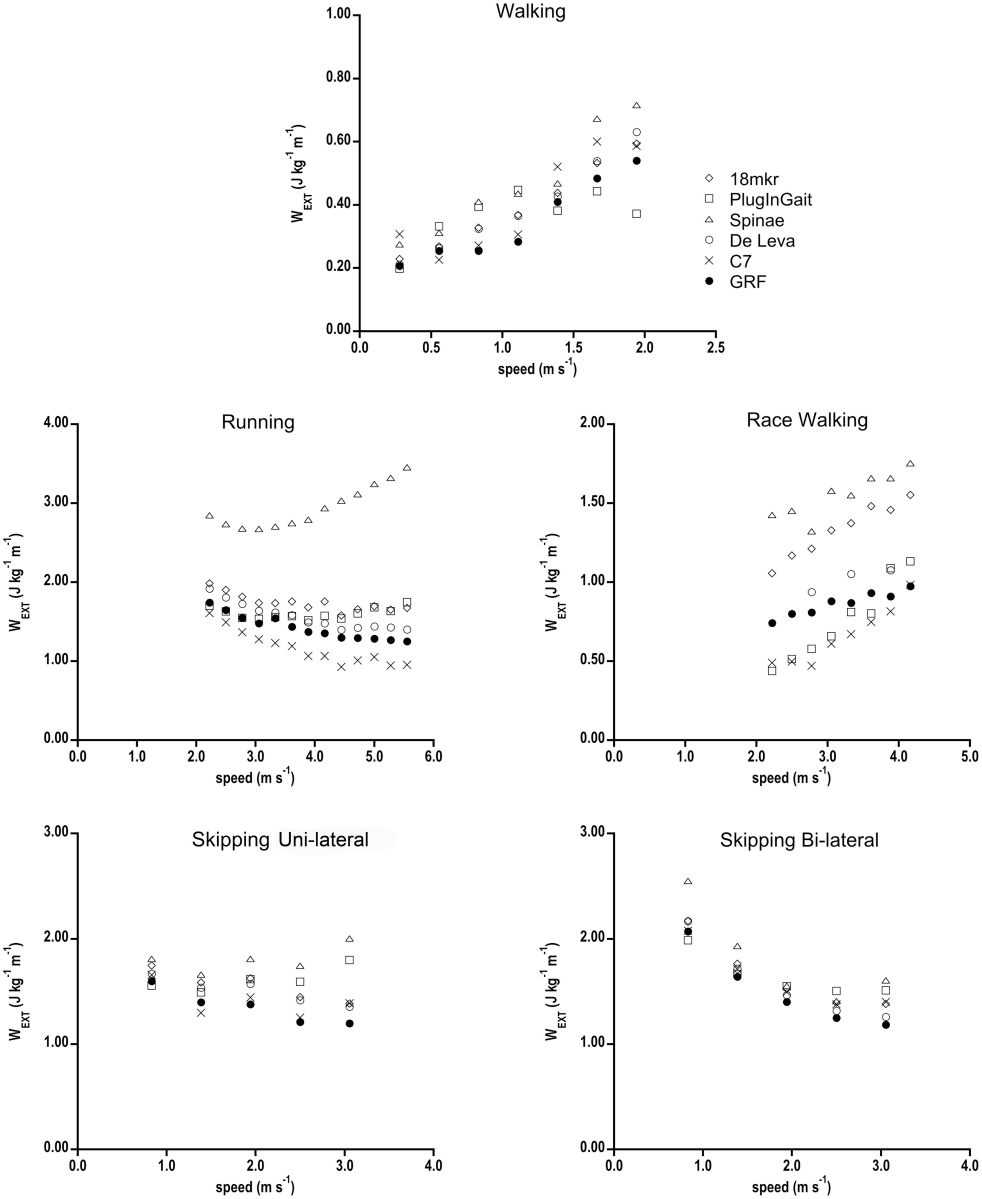
**W_**EXT**_ (J kg^**−1**^ m^**−1**^) of the 5 gaits as a function of speed calculated with the six different computational BCoM methods**. Standard deviations have been omitted for clarity.

W_EXT_ (J kg^−1^ m^−1^) as function of speed in all gaits is shown in Figure 3. In walking the overall trend of ID is an overestimation of FD values, with 18 mkr and De Leva marker sets better approaching GRF (Table 2). In running, Spinae mostly deviates from FD values, with De Leva and PlugInGait getting closest to them. In race walking, De Leva is close to GRF, while PlugInGait and C7 underestimate and 18 mkr and Spinae largely overestimates GRF. Bi-lateral Skipping was well-described by almost all ID methods, whereas in fast uni-lateral skipping no such consensus is reached.

**Figure 4:**
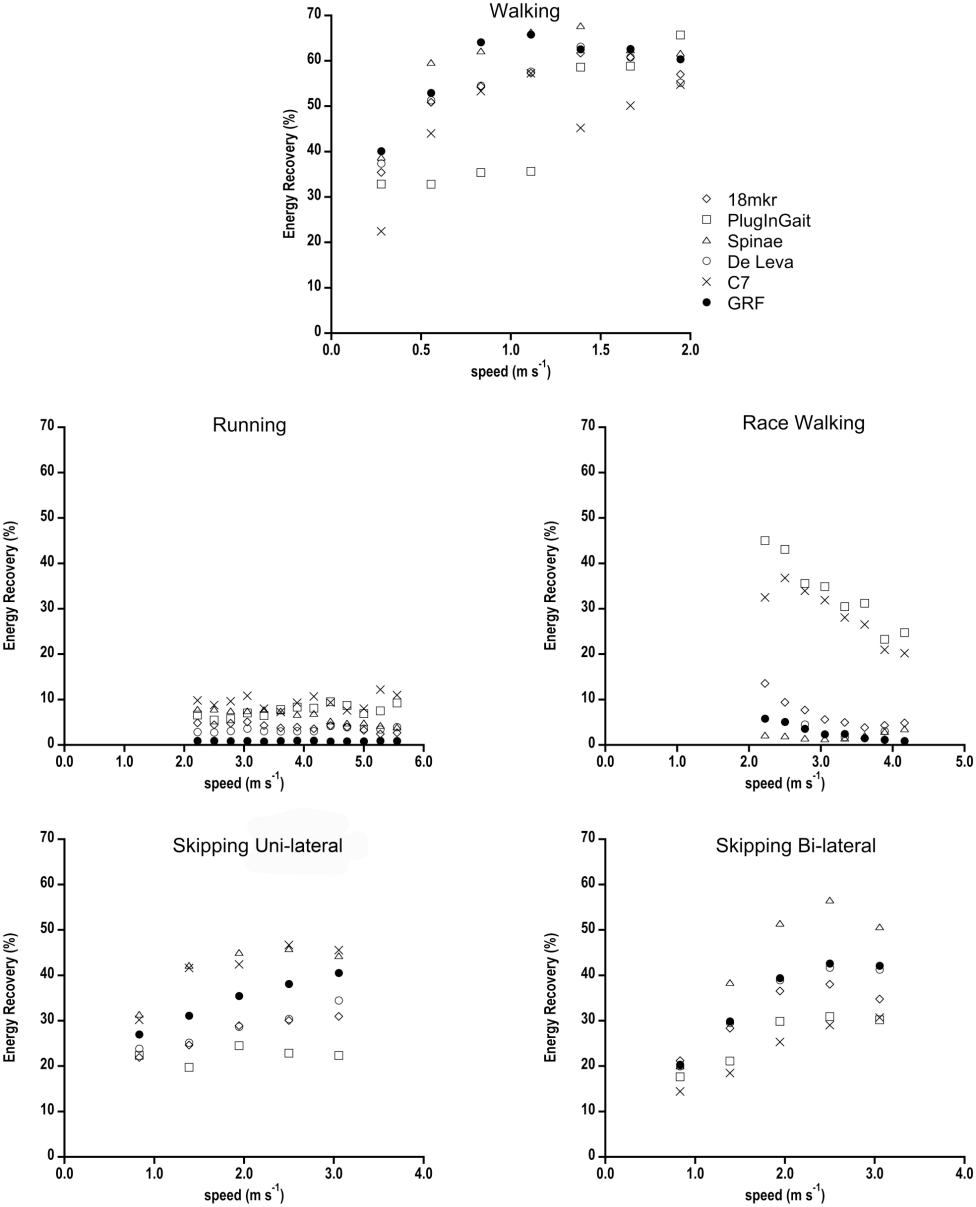
**Energy Recovery (%) as a function of speed calculated with the different computational BCoM methods, for all gaits**. Standard deviations have been omitted for clarity.

Energy Recovery (%) as function of speed in all gaits is shown in Figure 4. In walking the FD pattern is well-resembled by all ID methods apart from PlugInGait at low speeds and C7 at high speeds. Those two methods also reported the highest values in running (Table 2). In race walking, De Leva is the best predictor, Spinae and 18 mkr mostly match GRF data, whereas PlugInGait and C7 largely overestimate Energy Recovery values at all speeds. In uni-lateral skipping there is the major difference between Forward and Inverse Dynamics with a near 10% underestimation by De Leva and 18 mkr and overestimation of Spinae and C7. Conversely, in Bi-lateral skipping 18 mkr and De Leva match GRF data, Spinae still overestimates and C7 and PlugInGait underestimate FD results.

Statistical significance has been reached in the comparison between ID and FD values of W_EXT_ and Energy Recovery ID as, regardless the amount of absolute difference, the intra-stride analysis always found systematic changes (underestimation or overestimation) within a given condition (gait/speed).

**Figure 5:**
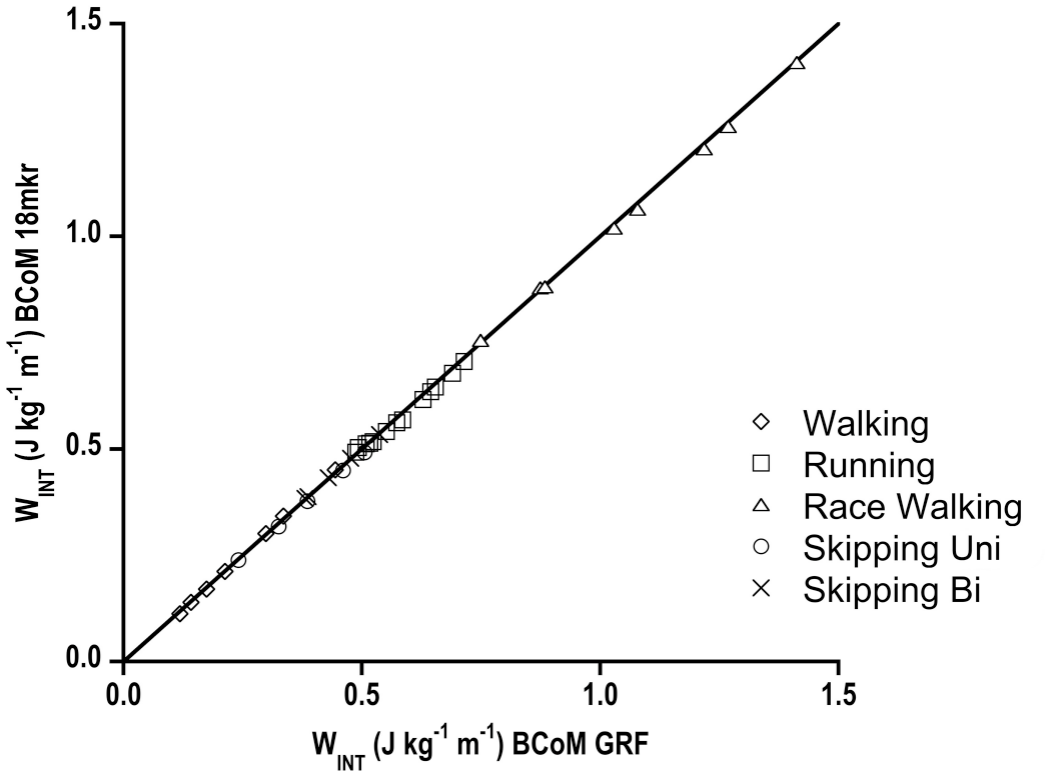
**W_**INT**_ (J kg^**−1**^ m^**−1**^) values where body segments speed was calculated with respect to Forward Dynamics BCoM speed (abscissa) vs. W_**INT**_ where their speed was obtained with respect to Inverse Dynamic BCoM speed (ordinate axis)**. Data are shown for the different gaits and continuous line represents identity line. Standard deviations have been omitted for clarity.

The W_INT_ obtained by calculating the kinetic energy of segments from their speed with respect to “true” BCoM (FD, GRF) is plotted in Figure 5 vs. W_INT_ computed with ID (18 mkr) BCoM as the reference, together with the identity line. This graph was arranged to check whether a compound variable depending, also, on the location of BCoM could be affected by ID methods. Race walking values are, as in walking and running, speed dependent, and almost two times higher than running at the same speed.

## Discussion

The aim of this paper was to compare Inverse to Forward Dynamics over the same paired stride (and a consistent amount of strides for each condition) in an extended range of speeds and human gaits regarding the ability to generate reliable 3D dynamical trajectories of BCoM. In the following, FD will be referred to as the “golden standard” toward which all ID methods should tend. Though the rationale of such a choice is straightforward, as discussed in the Introduction, it has to be considered that the current FD data processing, because of the peculiar experimental setup, included intensive filtering to eliminate the inevitable treadmill frame and engine vibrations transmitted to the underneath force sensors. This could have slightly smoothed the “reference” force signal.

The comparison started with “geometrical” or “spatial” parameters. The 3D RMS, a sort of average 3D distance between two trajectories, shows that two full body models, 18 mkr and De Leva, even based on (slightly) different anthropometric tables, consistently fitted the reference (FD) BCoM trajectory in walking, running and skipping (uni- or bi-lateral), differently from one marker placed on the trunk (C7) or the mean of pelvis (Spinae). In other words, 18 mkr and De Leva display the least inaccuracy. Again, when compared to other marker sets, in most gaits their values show some speed independency, reflecting a systematic error that can be associated to an inaccurate but precise estimate of the trajectory. These results, higher 3D RMS and speed dependency of simplistic models, highlight that it is indispensable for ID methods to include upper and lower limbs for describing the real (3D) BCoM trajectory. In addition, using full body models (18 mkr and De Leva), the 3D RMS value among gaits is very similar (see Table 2), and this strongly support the idea that the human body can be safely considered as a rigid multi-segment object in all gaits but race walking.

Our 3D RMS results in walking are comparable with Whittle (1997) and Eames et al. (1999) values, where center of pelvis overestimates BCoM trajectory when compared to both GRF and a whole body kinematic model (Vicon Body Builder). Gutierrez-Farewik et al. (2006) using Plug-In Gait marker set found a greater RMS than in this study. However, they validated the model by referring major discrepancies with respect to GRF method (mass evaluation error, integration speed constant in vertical axis and the constant speed assumption for antero-posterior axis). The present study has been designed to provide results from analysing an average of 65 strides for each gait/speed condition, not just a few of them as in Gutierrez-Farewik et al. (2006) on walkway platforms. By having obtained more accurate and precise gait parameters for that reason, a large 3D RMS value for Plug-In Gait marker set has here to be considered as an index of inaccuracy. As for running, even focusing on vertical axis only, Spinae never matches whole body model, as suggested by Gullstrand et al. (2009).

It comes with no surprise that lowest 3D RMS is observed for walking. Differently from all the other “bouncing gaits,” walking is supposed to have the least amount of wobbling of abdominal and muscle mass, which is one of the most bias of ID with respect to FD methods.

**Figure 6:**
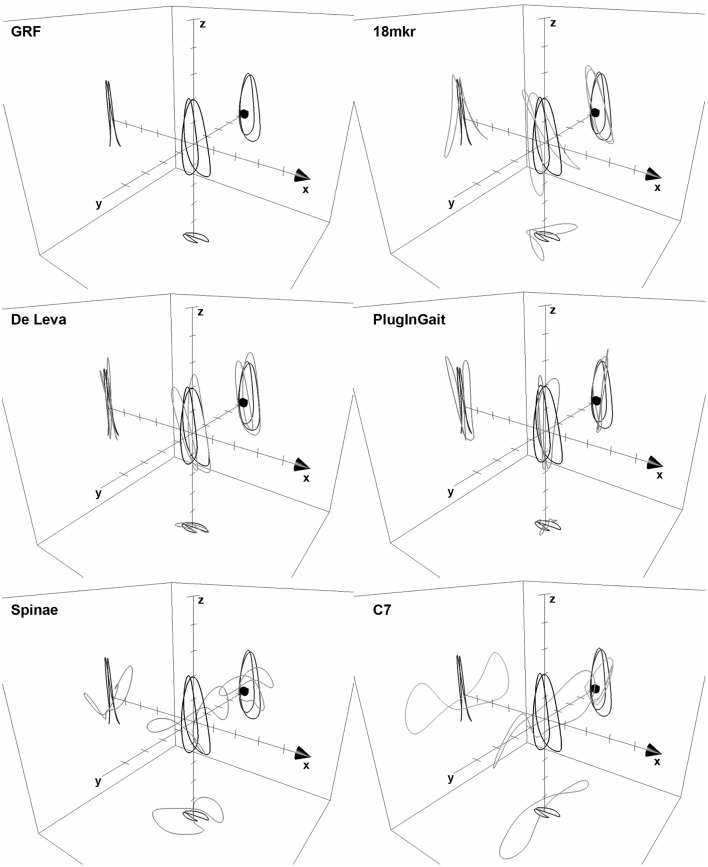
**The 3D BCoM Lissajous contour of the mean race walking stride at 3.89 m s^**−1**^, as obtained from FD and 5 ID methods is shown; arrow on x-axis indicates progression direction, axes ticks at 0.01 m step increment**. 3D BCoM contour from GRF is represented as a thick line in each panel for comparison.

**Figure 7:**
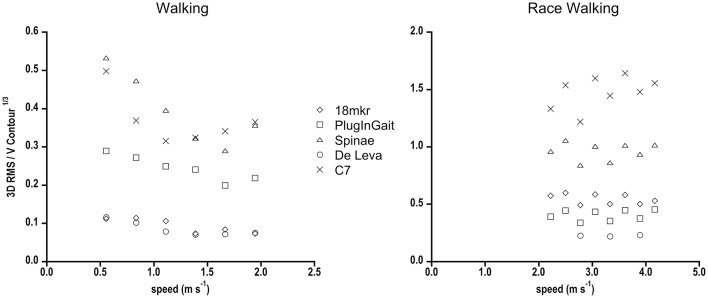
**3D RMS normalized for the cubic root of the 3D BCoM contour volume in walking and race walking as a function of speed, for the different Inverse Dynamics methods is shown**. Standard deviations have been omitted for clarity.

Since 3D RMS of race walking strides was comparable to the other bouncing gaits, despite of the remarkable inspectional discrepancy from FD in 3D BCoM trajectory as computed by (even the best approximating) ID methods (Figure 6), we standardized its 3D RMS for the contour volume (see Section Materials and Methods), as space occupancy of race walking contours is much smaller than in the other gaits (e.g., about eight times smaller, in volume, than walking). This should avoid disregarding a small (absolute) 3D RMS discrepancy, actually remarkable when occurring in a small contour. Figure 7 shows that dimensionless 3D RMS is larger in race walking (about 3x, compared to walking). Hence, race walking is the gait mostly suffering from Inverse Dynamics bias, regardless of the methodology. The reasons of such an error have to be found in race walking kinematics, where it is evident that the trunk does not act like as a rigid segment, with remarkable length changes in all three planes during the whole stride cycle (Pavei and La Torre, 2016). Models including the trunk as a single segment, e.g., 18 mkr, provide biased BCoM trajectory for this gait. However, even when the trunk is split in three parts and the model refined, as in De Leva, the trajectory becomes closer to Forward Dynamics (3D RMS) but its shape still does not resemble GRF (Figure 6).

The next step in the comparison between ID and FD techniques deals with the dynamics of travel of BCoM along its trajectory, i.e., with the ability to accurately reproduce the real speeds/accelerations during the path. The related variables are the kinetic energy, on the three axes, and, consequently, the positive work done by muscles to accelerate and raise the BCoM (W_EXT_).

In all gaits, the ID models differently under/overestimated FD W_EXT_ (Table 2). In walking, the greatest deviation from GRF (37%) was displayed by Spinae model; as walking is the most investigated gait by pathophysiology of locomotion, the use of such a simple model should be discouraged as the bias in total mechanical work and motion efficiency, which depends on W_EXT_, could reach unacceptable values. In running, Spinae is the most inaccurate marker set, whereas full body models only slightly overestimated W_EXT_ values. The overestimation of full body models primarily is due to the total energy fluctuation during flight phase. GRFs during flight are zero and then inherently predict a constant total mechanical energy during flight. The ID methods, instead, shows some fluctuations probably caused by undetectable wobbling masses (visceral and muscle mass) and body segment movement (also attributed to the misalignment between joint centers and skin markers). The increases in total energy (due to the fluctuations) cause, by definition, a higher amount of W_EXT_. Despite the difference in trajectory, only De Leva well-matched W_EXT_ data of race walking, whereas 18 mkr, Spinae, C7, and PlugInGait data are far from GRF values.

For all gaits but race walking, W_EXT_ overestimation of pooled 18 mkr and De Leva data with respect to GRF is quite constant in the whole range of speeds (about +13 ± 5% *p* < 0.01, with a range of 5–23%, see Table 2, in which race walking was not included for the quoted issues related to its BCoM contour). Such a uniform bias, due to the quoted issues about body anatomy and processing techniques [smoothing effects as signal filtering (FD and ID) and integration (FD); sharpening effects as differentiation (ID)], could be handy when comparing results from ID and FD studies of the same locomotor condition. Therefore, the best ID techniques can be considered, as mentioned, inaccurate but precise estimators of the dynamics of the BCoM.

As expected from the literature (Willems et al., 1995; Minetti, 1998a), Energy Recovery showed its maximal values in walking, minimal in running, and race walking, whereas skipping had intermediate values, slightly higher for its Bi-lateral variety. The analyzed models show various differences from GRF and in some cases (e.g., C7) also different trends among similar gaits. The heterogeneous response to different gait/speed conditions of an index reflecting the exchange between potential and kinetic energies of BCoM is here partly justified (as above) by the flight-induced bias of ID methods, an effect particularly evident in bouncing gaits. It has to be noted that the apparently huge (ID-FD)/FD values (%), shown in Table 2, reflect an almost normal variability about quantitatively small energy recovery values. As emerging from the discussion so far, the single marker model that functionally corresponds in modern times to the IMU (inertial measurement units, like activity monitors and smart phone apps) cannot still compete in term of spatial and temporal accuracy and precision with multi segment 3D kinematics and platform dynamometry.

The gaits here presented have been extensively studied in the past as far as the specific mechanical paradigm is concerned. From this point of view the energy recovery, a concept strictly related to the mechanical paradigm of the gait, could be considered the most sensitive variable in evaluating the appropriateness of a segmental method.

Generally speaking, race walking is the most problematic gait to be analyzed by ID methods. By considering all the investigated variables, De Leva seems to match previous literature studying race walking by means of GRF (i.e., Energy Recovery and W_EXT_, Cavagna and Franzetti, 1981), but still fails in overlapping the 3D BCoM trajectory of FD. The potential reason of the potential inaccuracy resides in the position of trunk markers, which better capture the movements mainly of the anterior part of the abdomen. Although De Leva represents the best choice among the investigated marker sets, its use should be recommended with caution. A new model, with a more comprehensive representation of the trunk conformational chances needs to be developed to study race walking biomechanics.

**Figure 8:**
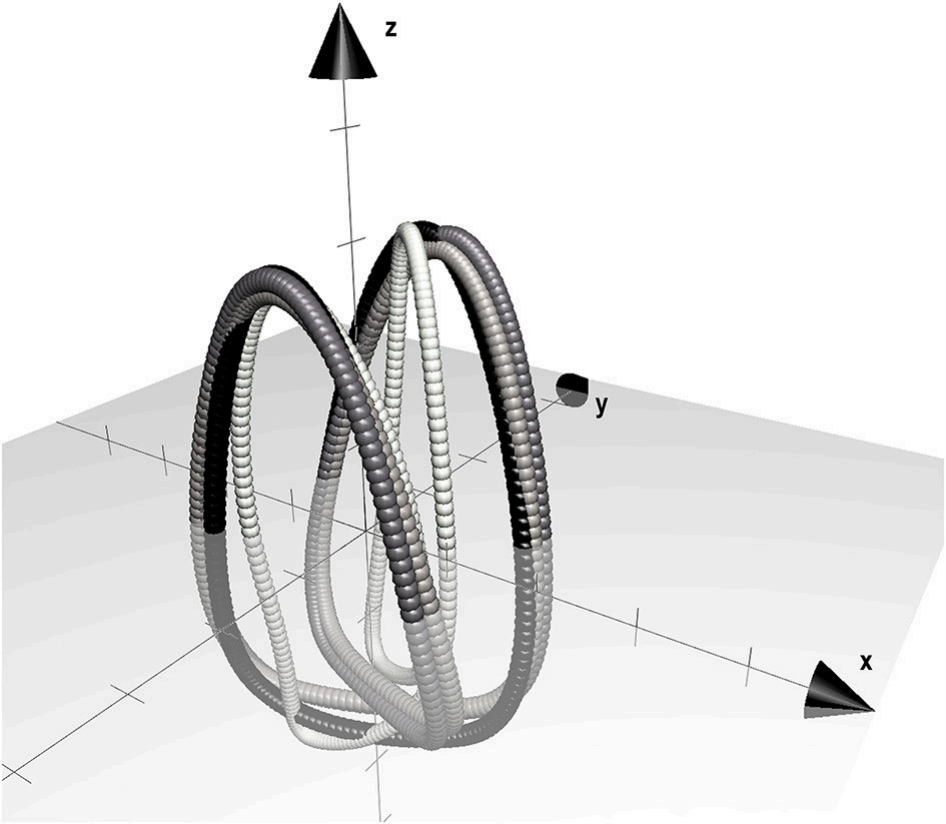
**3D BCoM trajectory of the mean walking stride at 1.67 m s^**−1**^ computed by three ID methods (18 mkr: dark gray, De Leva: light gray, PlugInGait: white) compared to FD (black)**. Arrow on x-axis indicates progression direction, axes ticks are located at 0.01 m step increment.

PlugInGait is a factory-made segmental model, being part of the Nexus Suite (Vicon, Oxford Metrics, UK) and BCoM position is estimated by proprietary software algorithm. When compared to GRF, it resembles true BCoM trajectory in running, but poorly in all other gaits, particularly walking. Since the shape is so different in walking (compared to 18 mkr and De Leva, see Figure 8) we guess that the only possible explanation of this discrepancy is a biased calculation of hips joint centers, from which trunk lengths, center of mass position and, ultimately, the overall BCoM are computed. It has to be pointed out that the manufacturer explicitly warns users in Plug-In Gait Manual: “Please note that this center of mass algorithm has not been clinically tested, and may be misleading in some clinical situations.” Actually, only some authors (Gutierrez-Farewik et al., 2006) validated it (just the vertical coordinate) in a clinical protocol about walking children. The present results suggest that it could be misleading also in a non-clinical environment.

As far as W_INT_ is concerned, it is clear that the small discrepancy between GRF and 18 mkr in BCoM trajectory does not affect W_INT_. Moreover, it is interesting to note that even when BCoM trajectory is totally far from GRF, as occurs in race walking, W_INT_ calculation is not biased. This validates the ID approach (no force plates) and potentially allows the estimation of W_INT_ also in race walking in comparison with other gaits.

This paper shows that the resemblance between Inverse and Forward Dynamics BCoM trajectories (and related parameters) strongly depends on the ID segmental model used. In general, documented multi-segment ID models provide a reliable estimate of 3D BCoM trajectory in many conditions. Among all the investigated gaits, race walking is the one in the need of an ideal segmental model, still to be designed.

There are potential limitations to this study.

Experimental protocol has been deliberately confined to just one subject. The core message of the present investigation is the pairwise discrepancy between ID- and FD-obtained 3D trajectories of the BCoM (and the related parameters), and not an evaluation of the adequacy of the subject's motion pattern in those (speed and gait) conditions. Actually, the presented values are in line with published data on the “general population” (e.g., Pavei et al., 2015). We can reasonably exclude that the obtained results reflect the individual characteristics of the only subject studied; even in case of someone with slight gait pathologies, it is likely that the same differences found here between pairs of methods would have hold.

All the considered marker sets are 3DOF because, differently from the joint moment approach, when the BCoM trajectory is calculated it seems not necessary to include segmental rotations other than those occurring within the sagittal plane. Also, the mediolateral offset of joint markers, symmetrically occurring with respect to that plane, is supposed not to affect the estimate of the “true” BCoM position.

The methodological comparison illustrated in this paper applies also outside the human realm. Motion pattern of BCoM in terrestrial animals during locomotion have been studied both via FD (Heglund et al., 1982; Genin et al., 2010) and ID (Minetti et al., 1999; Biancardi et al., 2011). While it was compulsory to use ID to estimate the mechanical internal work, which needs the position of segments with respect to BCoM, kinematics on treadmills have been preferred also in estimating BCoM with fast animals, as horses, where an impractically high number of platforms would have sampled just a couple of strides. It has to be considered that, differently from FD, ID can be used only if inertia parameters of body segments is known in advance. This is almost always viable in humans (unless we face body proportion widely deviating from the “standard” anthropometric tables, as in obese and pregnant subjects) but can be a real challenge with each investigated species, where only a few studies (if any) about segments inertia have been published.

Based on the present results, we conclude that among the investigated ID techniques, quite diffused in gait analysis laboratories as they also reveal limbs motion and the absolute height of BCoM, 18 mkr and De Leva marker sets provide the best approximation of FD BCoM trajectory, W_EXT_ and Energy Recovery in all gaits but race walking.

Future steps toward even better estimates from ID methods reside in the whole body determination of inertial parameters of individual segments through body contour image processing, particularly useful when body geometry deviates from anthropometric tables, and more accurate methods to established the dynamics of wobbling mass.

## Author contributions

GP and AM conceived the study, GP and ES collected data, GP, ES, and DC analyzed data, GP and AM wrote the manuscript and all the authors revised the final manuscript.

### Conflict of interest statement

The authors declare that the research was conducted in the absence of any commercial or financial relationships that could be construed as a potential conflict of interest.
